# Lack of Neurosteroid Selectivity at δ vs. γ2-Containing GABA_A_ Receptors in Dentate Granule Neurons

**DOI:** 10.3389/fnmol.2020.00006

**Published:** 2020-01-23

**Authors:** Xinguo Lu, Charles F. Zorumski, Steven Mennerick

**Affiliations:** ^1^Department of Psychiatry, Washington University School of Medicine, St. Louis, MO, United States; ^2^Department of Neuroscience, Washington University School of Medicine, St. Louis, MO, United States; ^3^Taylor Family Institute for Innovative Psychiatric Research, Washington University School of Medicine, St. Louis, MO, United States

**Keywords:** hippocampus, GABA_A_ receptor, neurosteroid, allopregnanolone (3α5αP), phasic inhibition, tonic inhibition

## Abstract

GABA_A_ receptors mediate a large fraction of inhibitory neurotransmission in the central nervous system. Two major classes of GABA_A_ receptors are γ2-containing receptors and δ-containing receptors, which are largely located synaptically and extrasynaptically, respectively. Neuroactive steroids such as allopregnanolone (3α5αP) and allotetrahydrodeoxycorticosterone (THDOC) are hypothesized to selectively affect δ-containing receptors over γ2-containing receptors. However, the selectivity of neurosteroids on GABA_A_ receptor classes is controversial. In this study, we re-examined this issue using mice with picrotoxin resistance associated with either the δ or γ2 subunit. Our results show that 3α5αP potentiated phasic inhibition of GABA_A_ receptors, and this is mainly through γ2-containing receptors. 3α5αP, with or without exogenous GABA, potentiated tonic inhibition through GABA_A_ receptors. Surprisingly, potentiation arose from both γ2- and δ-containing receptors, even when a δ selective agonist THIP was used to activate current. Although ethanol has been proposed to act through neurosteroids and to act selectively at δ receptors, we found no evidence for ethanol potentiation of GABA_A_ receptor function at 50 mM under our experimental conditions. Finally, we found that the actions of pentobarbital exhibited very similar effects on tonic current as 3α5αP, emphasizing the broad spectrum nature of neurosteroid potentiation. Overall, using chemogenetic analysis, our evidence suggests that in a cell population enriched for δ-containing receptors, neurosteroids act through both δ-containing and non-δ-containing receptors.

## Introduction

GABA_A_ receptors are ligand-gated, chloride-permeable ion channels mediating inhibitory neuronal transmission. They play essential roles in regulating neuronal activity and behavior (Sigel and Steinmann, 2012; Lee and Maguire, 2014). As heteromeric pentamers (Pirker et al., 2000), they are sometimes divided into two major classes: synaptically located γ2-containing receptors (γ2 receptors) and extrasynaptically located δ-containing receptors (δ receptors) (Wei et al., 2003). Developing subunit-selective drugs is a major goal of the pharmaceutical industry (Möhler, 2006, 2011); specifically δ selective drugs may have important applications in neuropsychiatry(Orser, 2006). For instance, neurosteroids have recently generated enthusiasm as antidepressants (Kanes et al., 2017; Gunduz-Bruce et al., 2019). Their psychoactive profile could result from selective effects on δ GABA_A_ receptors (Spigelman et al., 2003; Stell et al., 2003; Carver and Reddy, 2016), but some studies suggest limited selectivity (Shu et al., 2012). Here we reinvestigate neurosteroid subunit selectivity in native receptors with new tools in a well-studied class of δ-subunit expressing neurons.

Neurosteroids are one example of a wide variety of clinically important positive allosteric modulators of GABA_A_ receptors. Other positive modulators include benzodiazepines, barbiturates, and ethanol (Olsen et al., 2007; Olsen, 2018; Reddy, 2018; Masiulis et al., 2019). Ethanol and neurosteroids are proposed to share δ selectivity (Wallner et al., 2003; Wei et al., 2004; Olsen et al., 2007). In fact, ethanol’s positive effects on δ GABA_A_ receptor function may be mediated by altered endogenous neurosteroid production and/or release (Sanna et al., 2004; Tokuda et al., 2011; Izumi et al., 2015). Benzodiazepines are dependent on both the specific α subunit and γ subunit composition of receptors (Pritchett et al., 1989), and barbiturates are broad spectrum modulators (Olsen, 2018).

Neurosteroids prolong the GABA_A_ receptor response to synaptic GABA (IPSCs) and increase tonic GABA current (Stell et al., 2003). A prevalent view is that γ2 receptors are responsible for phasic inhibition, and δ receptors (in neurons that express the δ subunit) are responsible for tonic current, sometimes along with α5/γ2 receptors (Stell et al., 2003; Glykys et al., 2008; Brickley and Mody, 2012; Whissell et al., 2015). Previous studies are equivocal on whether the effects of neurosteroids on IPSCs are through γ2 or through δ receptors (Spigelman et al., 2003; Stell et al., 2003). Our own previous work suggests that slow, δ driven IPSCs are prominent in dentate granule cells (Sun et al., 2018) and so might explain neurosteroid effects. Tonic current is thought to arise largely (though not exclusively) from high-affinity δ receptors in cells that express δ subunits (Stell et al., 2003; Glykys et al., 2008; Brickley and Mody, 2012; Whissell et al., 2015), so δ-mediated tonic current also offers a potentially important substrate for δ-selective neurosteroid actions.

Previous studies employed genetic deletion to test neurosteroid selectivity (Stell et al., 2003; Glykys et al., 2008; Carver and Reddy, 2016), in part because of the lack of pharmacological tools to separate δ receptors from γ2 receptors. Although genetic deletion is typically considered more definitive than pharmacology, knockouts and knockdowns have caveats of compensation or other secondary changes that might affect outcomes (Gunther et al., 1995; Korpi et al., 2002; Peng et al., 2002). Further, sensitivity is sacrificed without the ability to measure pharmacological actions on different receptor populations in the same cell.

We recently revisited the role of δ receptors in phasic and tonic transmission using a knock-in/chemogenetic approach. We generated mice with picrotoxin (PTX) resistance associated with either the γ2 subunit (γ2^∗^ KI) or δ subunit (δ^∗^ KI) (Gurley et al., 1995; Sedelnikova et al., 2006). This strategy confers picrotoxin resistance onto receptors containing only a single mutated subunit (Gurley et al., 1995; Sun et al., 2018). Here we employed the new mouse lines to reinvestigate the selectivity of neurosteroids for the two main classes of GABA_A_ receptors in DGCs.

Our results showed that 3α5αP potentiated phasic inhibition by prolonging the decay of sIPSCs through an effect on γ2 receptors. 3α5αP also potentiated GABA_A_ tonic inhibition in slices from WT and the two knock-in genotypes. Based on complementary PTX sensitivity in the two mutants, the tonic current was mediated approximately equally by δ receptors and γ2 receptors. Furthermore, the potentiation of tonic current by neurosteroids persisted under conditions of no added agonist, when co-applied with exogenous GABA, and when co-applied with the δ-selective exogenous agonist THIP. We conclude that neurosteroids are not selective for δ receptors and that selectivity is unlikely to underlie unique psychoactive effects.

## Materials and Methods

### Slice Preparation

Mice from postnatal (P)25 to P32 GABA_A_ receptor δ^∗^ KI, γ2^∗^ KI, or WT littermates of both sexes were used (Sun et al., 2018). Mice were anesthetized with isoflurane and decapitated according to protocols approved by the Washington University IACUC. After attaching to a Leica VT1200 specimen holder with cyanoacrylate, coronal brain slices, 300-μM-thick, were cut in ice-cold, modified artificial CSF (aCSF; used for slicing in mM: 87 NaCl, 75 sucrose, 25 glucose, 25 NaHCO_3_, 2.5 KCl, 1.25 NaH_2_PO_4_, equilibrated with 95% oxygen-5% CO_2_ plus 0.5 CaCl_2_, 3 MgCl_2_; 320 mOsm). Slices recovered in choline-based ACSF (in mM: 92 choline chloride, 25 glucose, 30 NaHCO_3_, 2.5 KCl, 1.2 NaH_2_PO_4_, 20 HEPES, 2 thiourea, 5 Na ascorbate, 3 Na pyruvate, 2 CaCl_2_, and 1 MgCl_2_, equilibrated with 95% oxygen-5% CO_2_; 300 mOsm) at 32°C for 30 min. After recovery, slices were stored in regular aCSF (in mM: 125 NaCl, 25 glucose, 25 NaHCO_3_, 2.5 KCl, 1.25 NaH_2_PO_4_, equilibrated with 95% oxygen-5% CO_2_ plus 2.6 CaCl_2_, 1.2 MgCl_2_; 310 mOsm) for at least 1 h at 25°C before experimental recording. Drugs were obtained from Thermo Fisher Scientific except where noted.

### Whole-Cell Recording

Slices were transferred to a recording chamber with continuous perfusion (2 ml/min, 32°C) of oxygenated, regular aCSF. To measure phasic and tonic inhibition of GABA_A_ receptors, 10 μM NBQX (Tocris Bioscience) and 50 μM D-APV (Tocris Bioscience) were added in the regular aCSF to inhibit ionotropic glutamate receptors. To isolate current generated by the respective δ or γ2 subpopulation of GABA_A_ receptors, we applied 50 μM PTX (Tocris Bioscience) in the appropriate subunit knock-in tissue.

During somatic, whole-cell recording, hippocampal DGCs were visualized and identified by IR-DIC microscopy (Nikon FN1 microscope and Photometrics Prime camera). Borosilicate glass pipettes (World Precision Instruments, Inc.) with open tip resistance of 3–7 MΩ were used for whole-cell recording. Pipettes were filled with internal solution containing the following (in mM): 130 CsCl, 10 HEPES, 5 EGTA, 2 MgATP, 0.5 NAGTP, and 4 QX-314; pH adjusted to 7.3 with CsOH; 290 mOsm). A 5-min stabilization period was given after the whole-cell configuration was established, and then cells were recorded with a MultiClamp 700B amplifier (Molecular Devices), Digidata 1550 16-bit A/D converter, and pClamp 10.4 software (Molecular Devices).

### Measurement of Phasic and Tonic GABA_A_ Receptor Current

Cells were voltage-clamped at −70 mV during whole-cell recordings. Spontaneous phasic GABA_A_ receptor currents were measured before/after the application of 3α5αP (100 nM). Tonic GABA_A_ receptor currents were activated by GABA (5 μM), THIP (1 μM), and/or 3α5αP (100 nM). In some experiments, DS2 (Tocris Bioscience) and allotetrahydrodeoxycorticosterone (THDOC) were used as comparators. PTX was added at the end to isolate δ^∗^ KI or γ2^∗^ KI-mediated GABA_A_ receptor currents. A 5-min perfusion of each drug was used to ensure maximal effect. All recordings were acquired in gap-free mode at 5 kHz, filtered at 2 kHz using an 8-pole Bessel filter.

### Analysis and Statistics

Both phasic and tonic GABA_A_ currents were measured after cells reached steady state for each condition. sIPSCs and tonic currents were measured as described previously (Sun et al., 2018). At least 40 events contributed to average sIPSC waveforms. sIPSC decays were fit to the sum of two exponential functions, extrapolated to the peak IPSC. Decay time course is reported as a weighted time constant (τ_w_) (Sun et al., 2018). Tonic GABA_A_ currents were obtained by subtracting baseline holding currents. A paired *t* test or one-way repeated-measures ANOVA was performed using GraphPad Prism 8 to detect the effects within cells. Specific analyses are described in the Results. Significance is presented at the level of *p ≤ 0.05, **0.01, ***0.001, and ****0.0001. Summary data are presented as mean ± SEM.

## Results

### Potentiation of Phasic Inhibition by 3α5αP

**FIGURE 1:**
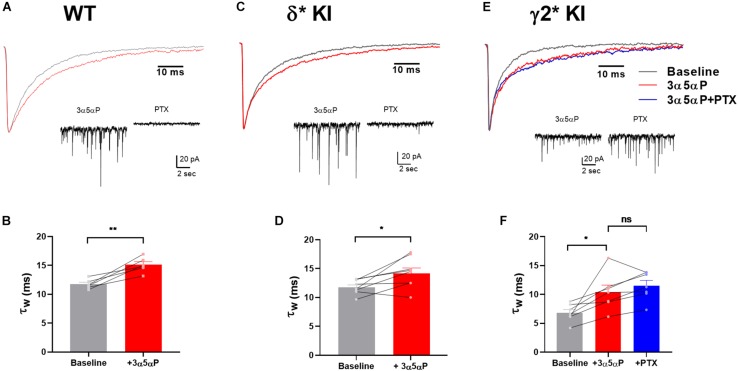
Prolongation of sIPSC decay by 3α5αP in hippocampal DGCs. **(A)** Representative WT average sIPSC waveforms obtained before (black) and in the presence (red) of 100 nM 3α5αP. **(B)** WT DGC τ_w_ of sIPSC decay under baseline (11.7 ± 0.3 ms) and 3α5αP (15.1 ± 0.5 ms). **(C)** δ* KI average sIPSC waveforms obtained before, in the presence of 100 nM 3α5αP. **(D)** δ* KI DGC τ_w_ of sIPSC decay under baseline (11.9 ± 0.4 ms) and 3α5αP (14.3 ± 0.9 ms). **(E)** γ2* KI average sIPSC waveforms obtained before, in the presence of 100 nM 3α5αP, and with addition of 50 μM PTX. **(F)** γ2* KI DGC τ_w_ of sIPSC decay under baseline (6.8 ± 0.6 ms) and 3α5αP (10.4 ± 1.2 ms), and 3α5αP/PTX (11.5 ± 0.9 ms). Paired *t* test showed that 100 nM 3α5αP significantly increased τ_w_ of sIPSC decay in WT (*n* = 6, *p* = 3 × 10^–3^) and δ* KI (*n* = 8, *p* = 0.02). In γ2* KI (*n* = 7) one-way ANOVA on τ_w_ of sIPSC decay showed a drug effect (*F*(1.3,7.7) = 15.3, *p* = 4 × 10^–3^), with a significant difference between baseline and 3α5αP (Holm-Sidak, *p* = 0.04), and no change between 3α5αP and PTX (Holm-Sidak, *p* = 0.2). The τ_w_ of sIPSC decay after PTX was not included in panels **(B,D)** due to the elimination of most sIPSCs by PTX.

Neurosteroids like 3α5αP and THDOC potentiate phasic inhibition by prolonging the decay of sIPSCs in hippocampal DGCs (Stell et al., 2003). Although this effect has previously been associated with γ2 receptors, neurosteroids at sufficiently modest concentrations may have selective effects on δ receptors (Stell et al., 2003; Carver and Reddy, 2016), which may participate more prominently in phasic transmission in DGCs than typically appreciated (Spigelman et al., 2003; Sun et al., 2018). In WT cells, 100 nM 3α5αP prolonged the weighted time constant (τ_w_) of sIPSC decay (Figures 1A,B). In δ^∗^ KI DGCs, 3α5αP showed a similar prolongation of sIPSC decay (Figures 1C,D). PTX eliminated most sIPSCs in both WT and δ^∗^ KI DGCs (Figures 1A,C). In γ2^∗^ KI DGCs, 100 nM 3α5αP also prolonged the τ_w_ of sIPSC decay (Figures 1E,F). However, baseline γ2^∗^ sIPSC decay was faster than the other genotypes, as previously reported (Sun et al., 2018). After PTX addition, the prolongation of sIPSC decay persisted in γ2^∗^ cells (Figures 1E,F). Taken together, these results indicate that 3α5αP prolongs the decay of sIPSCs in DGCs, and this potentiation is mainly through γ2 receptors. These results also confirm observations of others (Stell et al., 2003) but are in contrast with results suggesting a large impact of neuroactive steroids on a δ component of sIPSCs (Spigelman et al., 2003).

Although the major effect of neurosteroids is on IPSC time course, we also examined sIPSC frequency and amplitude. There was a trend toward decreased frequency with 3α5αP addition in each of the three genotypes: [WT, *n* = 6: 2.1 ± 0.5 Hz vs. 1.5 ± 0.2 Hz; δ^∗^, *n* = 8: 3.8 ± 0.7 Hz vs. 2.8 ± 0.5 Hz; γ2^∗^, *n* = 7: 2.7 ± 0.8 Hz vs. 2.2 ± 0.4 Hz; (*F*(1.0,18.0) = 6.7, *p* = 0.02); 2-way ANOVA, with repeated measures for frequency]. This likely reflects a general suppression of network activity by the pro-inhibitory neurosteroid. By contrast, there was no consistent effect of 3α5αP on amplitude of sIPSCs [WT: −38.9 ± 1.4 pA vs. −32.5 ± 2.0 pA; δ^∗^: −37.7 ± 2.5 pA vs. −39.9 ± 1.8 pA; γ2^∗^: −54.8 ± 11.9 pA vs. −50.1 ± 6.0 pA; (*F*(1.0,18.0) = 0.8, *p* = 0.4)].

**FIGURE 2:**
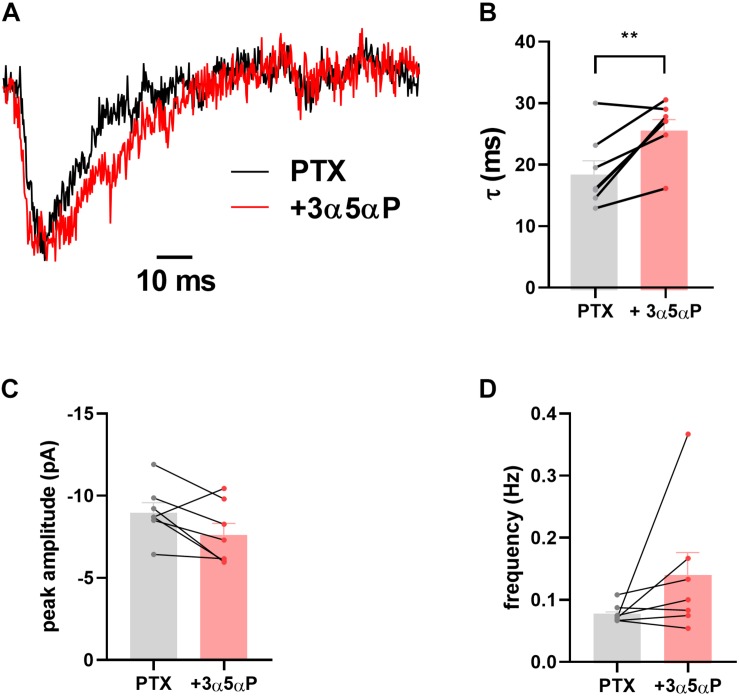
Prolongation of δ sIPSC decay. In the presence of 50 μM PTX δ sIPSCs were isolated as described (Sun et al., 2018). **(A)** Scaled sIPSC waveforms from baseline and in the presence of 100 nM 3α5αP from 15 and 17 events, respectively. **(B–D)** In δ* KI DGCs (*n* = 7) decays were prolonged by 100 nM 3α5αP from 18.9 ± 2.3 ms to 26.1 ± 1.8 ms (paired *t* test, *p* = 0.01). Neither sIPSC amplitude (−9.1 ± 0.6 pA vs. −7.7 ± 0.7 pA) nor frequency (0.08 ± 0.006 Hz vs. 0.14 ± 0.04 Hz) was reliably altered (paired *t* test, *p* = 0.08, 0.2, respectively).

We have previously shown that sIPSCs mediated by δ receptors are detectable in DGCs, albeit at a low frequency compared with γ2-dominated sIPSCs (Sun et al., 2018). In δ^∗^ slices incubated in 50 μM PTX, we found that 3α5αP similarly prolonged δ sIPSCs (Figure 2). Because of the small number of baseline δ sIPSCs detected, the overall impact on phasic transmission mediated by δ receptors was modest.

### Potentiation of Tonic Inhibition by 3α5αP Alone

**FIGURE 3:**
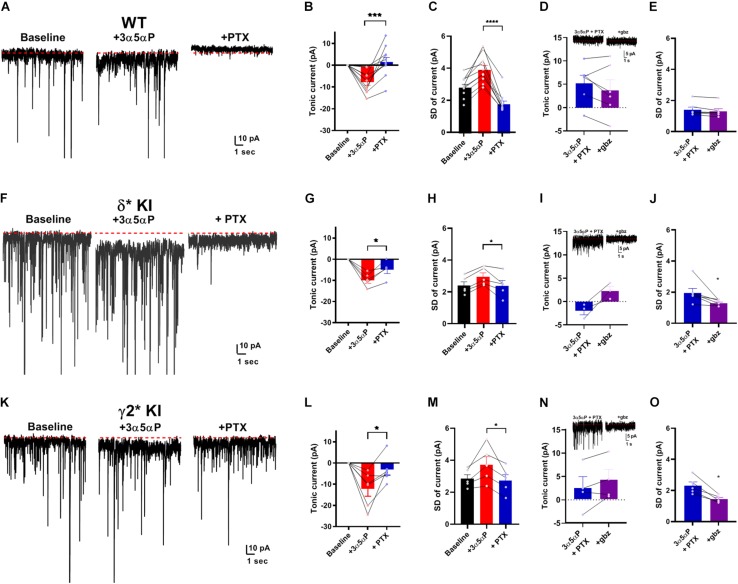
Potentiation of tonic current by 3α5αP alone. **(A)** Effect of 100 nM 3α5αP in a representative DGC from a WT slice. 3α5αP resulted in a tonic current that was blocked by 50 μM PTX. Note that the sIPSCs were also largely inhibited by PTX, as also shown in Figure 1. **(B)** Summary of changes in mean current relative to baseline. 3α5αP increased tonic current to −7.4 ± 1.5 pA, and PTX decreased it to 2.0 ± 2.2 pA. **(C)** Summary of the standard deviation of current (baseline: 2.9 ± 0.2 pA, 3α5αP: 4.0 ± 0.3 pA, and 3α5αP+PTX: 1.8 ± 0.2 pA). **(D)** In a subset of neurons, 50 μM gabazine (gbz) was tested and produced no clear further impact on tonic current beyond the effect of PTX. Inset shows representative current samples. **(E)** Gbz also produced minimal change in the standard deviation of the current, indicating that PTX fully inhibited tonic current. **(F–J)** The same experiment from δ* KI DGCs. 3α5αP increased tonic current from baseline to −9.6 ± 1.5 pA, and PTX decreased it to −4.6 ± 1.8 pA. Standard deviation of current for baseline, 3α5αP, and 3α5αP+PTX was 2.4 ± 0.3 pA, 3.0 ± 0.3 pA, and 2.4 ± 0.3 pA, respectively. **(K–O)** Results from γ2* KI animals. 3α5αP increased tonic current from baseline to -12.3 ± 3.4 pA, and PTX decreased it to −3.3 ± 2.5 pA. Standard deviation of current for baseline, 3α5αP, and 3α5αP+PTX was 2.9 ± 0.3 pA, 3.8 ± 0.5 pA, and 2.8 ± 0.4 pA, respectively. Paired *t* test showed significant difference of tonic currents between 3α5αP and PTX in WT (*n* = 10, *p* = 9 × 10^–4^), δ* KI (*n* = 5, *p* = 0.01), and γ2* KI (*n* = 6, *p* = 0.03). To compare genotype effects, we examined differences in the SD values prior to and during gabazaine (not directly shown; statistics performed on difference between connected SD values). An ANOVA between 3α5αP+PTX and 3α5αP+PTX+gabazine showed a genotype effect (*F*(2.0,14.0) = 4.2, *p* = 0.03), with both δ* (0.7 ± 0.3 pA, *n* = 6) and γ2* (0.9 ± 0.2 pA, *n* = 5) exhibiting a difference from WT (0.1 ± 0.03 pA, *n* = 6) (Holm-Sidak, *p* = 0.05, 0.03, respectively). For panels sample current traces in this and subsequent figures, some of the largest sIPSCs have been truncated for clarity of depiction of tonic current.

Given that neurosteroid effects on IPSCs do not appear to involve strong δ selectivity, we turned to tonic currents, where evidence more strongly supports selective δ receptor effects of neurosteroids. A prevalent view is that δ receptors mediate a strong tonic component in DGCs. The selectivity hypothesis predicts that potentiation of tonic inhibition by neurosteroids is mainly through δ receptors (Stell et al., 2003; Glykys et al., 2008; Carver and Reddy, 2016). Under similar conditions to Figure 1, we examined the effect of 3α5αP on tonic current. 3α5αP similarly potentiated a tonic current in all three genotypes [one-way ANOVA, (*F*(2.0,18.0) = 1.4, *p* = 0.3)]. In WT slices, both 3α5αP tonic current and phasic current were completely blocked by PTX (Figures 3A,B). In both δ^∗^ KI and γ2^∗^ KI DGCs, PTX partially inhibited the 3α5αP tonic current (Figures 3F,G,K,L).

Because the currents observed were very small, and because holding current may be affected by technical factors over time, we examined an alternate measure of GABA channel activity. Channel-mediated currents are characterized by fluctuations (noise) that result from the stochastic nature of channel opening and closing (Anderson and Stevens, 1973). At low probabilities of channel opening, current fluctuations should correlate with the tonic current (Farrant and Nusser, 2005; Lingle, 2006) As a simple measure of these current fluctuations, we calculated the root mean squared noise level (standard deviation after subtracting the mean current) in each experimental condition. Figures 3C,H,M show that the results of this analysis parallel results from measures of the mean current and are arguably more sensitive than changes in current amplitudes. In a subset of cells, we also tested the impact of gabazine co-applied following PTX addition. Results showed trends toward further reduction in the two mutant phenotypes with no change in WT (Figures 3D,I,N). Measures of SD were clearer and showed minimal effect of gabazine on fluctuations following PTX addition (Figure 3E) but reduced fluctuations in both δ^∗^ and γ2^∗^ backgrounds (Figures 3J,O). Taken together, the results demonstrate that in the absence of added agonist, both γ2 and δ receptors contribute to the effect of 3α5αP on tonic GABA currents.

### Potentiation of Tonic Inhibition by 3α5αP With Exogenous GABA

**FIGURE 4:**
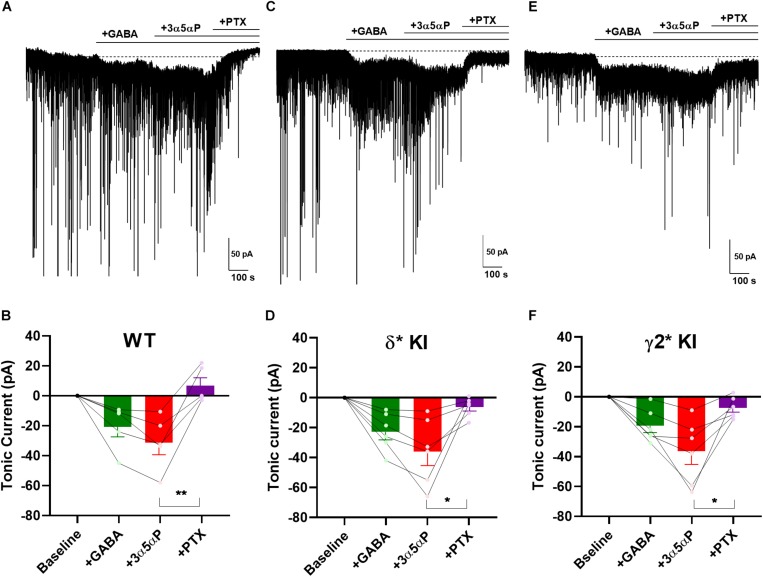
3α5αP potentiation of tonic inhibition with exogenous GABA is non-selective. **(A)** Effects of exogenous GABA (5 μM) in a representative DGC from a WT slice. GABA generated a current superimposed on ongoing spontaneous phasic sIPSCs. 3α5αP (100 nM) potentiated the GABA response, and 50 μM PTX abolished both the phasic and tonic currents. **(B)** Summary of the GABA/3α5αP current in the indicated conditions from cells of WT animals (*n* = 5). GABA produced a tonic current of −20.2 ± 6.7 pA, and 3α5αP increased it to −31.0 ± 7.8 pA, while PTX decreased the tonic current to 7.3 ± 5.4 pA. **(C,D)** The same experiment from δ* KI DGCs (*n* = 6). Similar as WT, GABA generated a tonic current of −22.6 ± 5.2 pA. Note that the GABA/3α5αP tonic current was partially blocked by PTX (from −35.6 ± 9.0 pA to −6.2 ± 2.6 pA), and phasic currents were strongly reduced. **(E,F)** Same experiment from γ2* KI DGCs (*n* = 6). Comparable to δ* KI slices, GABA activated a tonic current of -19.6 ± 4.6 pA. The GABA/3α5αP current was also partially blocked by PTX (from −36.6 ± 8.8 pA to −7.5 ± 2.9 pA), but the phasic currents were left mostly intact. Dotted line in panels **(A,C,E)** shows mean initial holding current, which was subtracted for pooled results in panels **(B,D,F)**. One-way ANOVA on tonic current showed a drug effect in WT (*F*(1.9,7.4) = 17.0, *p* = 2 × 10^–3^), δ* KI (*F*(1.3,6.4) = 11.3, *p* = 0.01), and γ2* KI (*F*(1.4,6.9) = 13.5, *p* = 0.02). PTX significantly decreased GABA/3α5αP current in WT (Holm-Sidak, *p* = 5 × 10^–3^), δ* KI (Holm-Sidak, *p* = 0.03), and γ2* KI (Holm-Sidak, *p* = 0.02).

Perhaps the near absence of ambient GABA, suggested by the small effect of PTX in WT slices and gabazine in mutant slices (Figure 3), is not physiological and therefore does not invoke selectivity that might otherwise be generated by neurosteroids. To ensure that neurosteroids have the opportunity to interact with agonist-bound receptors, we applied 5 μM GABA to produce a tonic current of about 30 pA (Figure 4) (Lee and Maguire, 2014). Note that the actual GABA concentration reaching cells is likely much lower, as a result of avid GABA transport by neurons and glia (Nusser and Mody, 2002; Jensen et al., 2003; Zhan and Nadler, 2009). 100 nM 3α5αP increased the tonic current, and PTX fully blocked this current (Figures 4A,B). In δ^∗^ KI DGCs, GABA/3α5αP produced a current of similar amplitude to WT that was partially sensitive to PTX (Figures 4C,D). This suggests that the 3α5αP-potentiated tonic current is only partially mediated by δ receptors in DGCs and a substantial fraction is mediated by γ2 receptors. Also, in γ2^∗^ KI DGCs, GABA/3α5αP generated a similar amount of tonic current as WT and δ^∗^ KI. A one-way ANOVA showed no difference among genotypes (*F*(2.0,14.0) = 0.1, *p* = 0.9). sIPSCs persisted in PTX, again consistent with γ2 receptors as the main drivers of phasic inhibition. Interestingly, PTX did not fully block the tonic current in γ2^∗^ slices (Figures 4E,F). The complementary results from δ^∗^ and γ2^∗^ KI DGCs suggests that 3α5αP potentiates tonic current arising from both γ2 and δ receptors.

### Potentiation of Tonic Inhibition by 3α5αP and THDOC With Subunit-Selective Agonist

**FIGURE 5:**
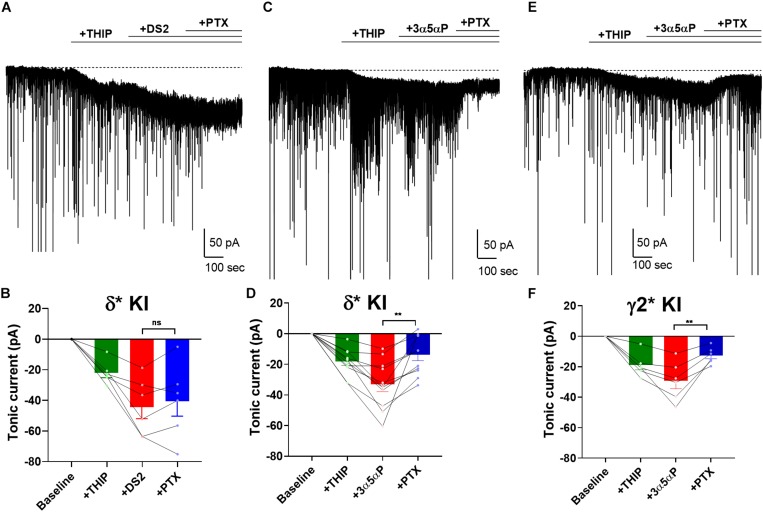
3α5αP potentiates THIP-generated current. **(A)** Effects of 1 μM THIP plus 10 μM DS2 in a representative DGC from a δ* KI slice. DS2 potentiated the THIP-evoked current, and this current was resistant to 50 μM PTX. **(B)** Summary of the current in the indicated conditions from cells of δ* KI animals (*n* = 6). Tonic current under THIP, THIP+DS2, and THIP+DS2+PTX was −21.9 ± 3.0 pA, −44.1 ± 7.6 pA, and −40.2 ± 9.8 pA, respectively. **(C)** Effects of THIP/100 nM 3α5αP in a representative DGC from a δ* KI slice. 3α5αP potentiated the THIP-generated current, but this current was partially blocked by PTX. **(D)** Summary of the THIP/3α5αP current in δ* KI DGCs (*n* = 11). Tonic current under THIP, THIP+3α5αP, and THIP+3α5αP+PTX was -18.4 ± 2.6 pA, −33.2 ± 4.8 pA, and -13.9 ± 3.9 pA, respectively. **(E,F)** Same experiment from γ2* KI DGCs (*n* = 6). Tonic current under THIP, THIP+3α5αP, and THIP+3α5αP+PTX was -19.1 ± 3.1 pA, −29.3 ± 5.2 pA, and -12.7 ± 2.2 pA, respectively. Note that the GABA/3α5αP current was also partially blocked by PTX, while the phasic currents were mostly left intact. One-way ANOVA on tonic current showed a drug effect in δ* KI THIP/DS2 (*F*(1.3,6.4) = 18.5, *p* = 3 × 10^–3^), δ* KI THIP/3α5αP (*F*(2.3,22.7) = 25.1, *p* < 1 × 10^–4^), and γ2* KI THIP/3α5αP (*F*(1.5,7.4) = 25.7, *p* = 7 × 10^–4^). PTX did not change the THIP/DS2 tonic current in δ* KI (Holm-Sidak, *p* = 0.4), however PTX significantly decreased THIP/3α5αP generated tonic current in both δ* KI (Holm-Sidak, *p* = 2 × 10^–3^), and γ2* KI (Holm-Sidak, *p* = 7 × 10^–3^).

Surprisingly, our results showed that 3α5αP potentiated both δ and γ2 receptors to produce/potentiate tonic inhibition. To determine whether selectivity can be increased by activating receptors with a low concentration of δ-preferring agonist, we employed THIP as agonist. Previously, we showed that 1 μM THIP plus 10 μM DS2 potentiated tonic inhibition in DGCs, and this drug combination yielded a current mainly through δ receptors in DGCs (Sun et al., 2018). To evaluate the selectivity of 3α5αP on GABA_A_ receptors when a δ-selective agonist is used, we co-applied THIP and 3α5αP to KI slices. As a positive control, we repeated our previous experiment using DS2, a positive allosteric modulator selective for δ receptors (Jensen et al., 2013). As expected, in δ^∗^ KI slices, the tonic current generated by THIP plus DS2 was resistant to PTX (Figures 5A,B). Although THIP/3α5αP resulted in a tonic current in δ^∗^ KI slices comparable in amplitude to the THIP plus DS2 current, this current was only partially inhibited by PTX, indicating a contribution from non-δ receptors (Figures 5C,D). THIP/3α5αP resulted in a similar tonic current in γ2^∗^ KI slices. Complementary to the result in δ^∗^ KI slices, this current was also partially blocked by PTX (Figures 5E,F).

**FIGURE 6:**
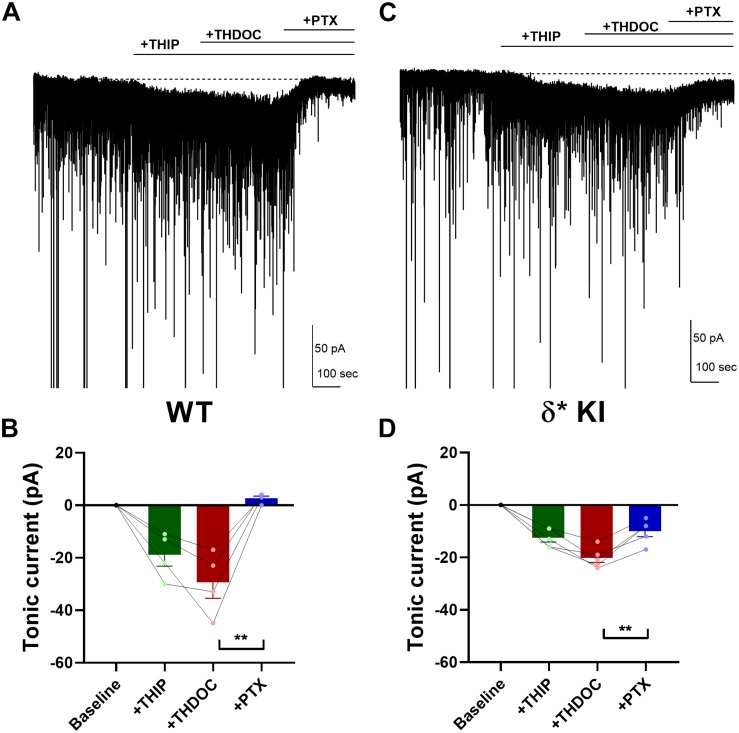
THDOC potentiates THIP-produced current. **(A)** Effect of 100 nM THDOC in a representative DGC from a WT slice. THDOC potentiated the THIP (1 μM) generated current, which was blocked by 50 μM PTX. **(B)** Summary of the current in the indicated conditions from WT DGCs (*n* = 4). Tonic current under THIP, THIP+THDOC, and THIP+THDOC+PTX was -19.0 ± 4.4 pA, −29.5 ± 6.1 pA, and 2.5 ± 0.9 pA, respectively. **(C,D)** Same experiment from δ* KI DGCs (*n* = 5). Note that the THIP/THDOC current was partially blocked by PTX. Tonic current under THIP, THIP+THDOC, and THIP+THDOC+PTX was -12.6 ± 1.6 pA, −20.2 ± 1.8 pA, and -10.0 ± 2.1 pA, respectively. One-way ANOVA on tonic current showed a drug effect in WT (*F*(1.6,4.8) = 22.9, *p* = 4 × 10^–3^), and δ* KI (*F*(2.7,10.6) = 42.9, *p* < 1 × 10^–4^). PTX significantly decreased THIP/THDOC tonic current in WT (Holm-Sidak, *p* = 0.01) and δ* KI (Holm-Sidak, *p* = 3 × 10^–3^).

To probe neurosteroid selectivity further, we employed another commonly used neurosteroid, THDOC. In WT slices, THDOC potentiated the THIP-generated tonic current, and this current was fully blocked by PTX (Figures 6A,B). Similar to THIP/3α5αP results, in δ^∗^ KI slices the THIP/THDOC tonic current was again only partially inhibited by PTX (Figures 6C,D), indicating the partial contribution of δ receptors to the tonic inhibition. Taken together, our results show that in contrast to DS2, either 3α5αP or THDOC co-applied with THIP acts on both δ and γ2 receptors to potentiate tonic currents.

### Lack of Potentiation of Tonic Inhibition by Ethanol

**FIGURE 7:**
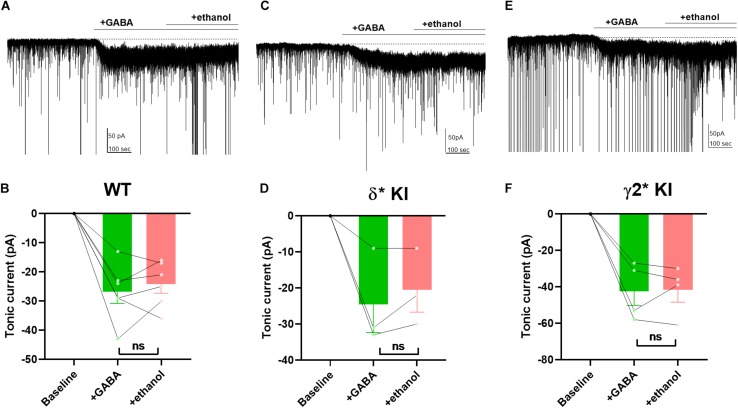
Lack of potentiation of tonic current by ethanol. **(A)** Effects of 50 mM ethanol after 5 μM GABA in a representative DGC from a WT slice. GABA activated a current superimposed on ongoing spontaneous phasic sIPSCs. Ethanol did not potentiate the tonic current. **(B)** Summary of the GABA/ethanol current in the indicated conditions from cells of WT animals. Tonic current activated by GABA and GABA+ethanol was −26.8 ± 4.0 pA and −24.2 ± 3.2, respectively. **(C,D)** Same experiment from δ* KI animals. Tonic current generated by GABA and GABA+ethanol was −24.3 ± 7.7 pA and −20.3 ± 6.1 pA, respectively. **(E,F)** Same experiment from γ2* KI animals. Tonic current produced by GABA and GABA+ethanol was −42.3 ± 7.8 pA and −41.5 ± 6.8 pA, respectively. Paired *t* test showed no difference of the tonic currents between GABA and ethanol in WT (*n* = 6, *p* = 0.4), δ* KI (*n* = 3, *p* = 0.3), and γ2* (*n* = 4, *p* = 0.9).

Ethanol has been shown to potentiate GABA_A_ receptors by enhancing a tonic current through δ receptors (Wallner et al., 2003; Wei et al., 2004; Olsen et al., 2007). However, other studies suggest that ethanol has no effect on GABA_A_ currents, or that potentiation may simply depend on ambient GABA concentration (Borghese et al., 2006; Yamashita et al., 2006; Fleming et al., 2011). Thus, it remains unclear and controversial whether ethanol directly targets GABA_A_ receptors. To investigate the effects of ethanol on GABA_A_ receptors, we applied 50 mM ethanol with 5 μM GABA to hippocampal DGCs. Despite robust GABA-generated currents (Figures 7A–F), ethanol failed to potentiate the current in any of the three genotypes (Figures 7B,D,F). Our results show that in contrast to 3α5αP, ethanol does not target GABA_A_ receptors responsible for GABA-generated tonic current in DGGs under these conditions.

### Potentiation of Tonic Inhibition by Pentobarbital With Exogenous GABA

**FIGURE 8:**
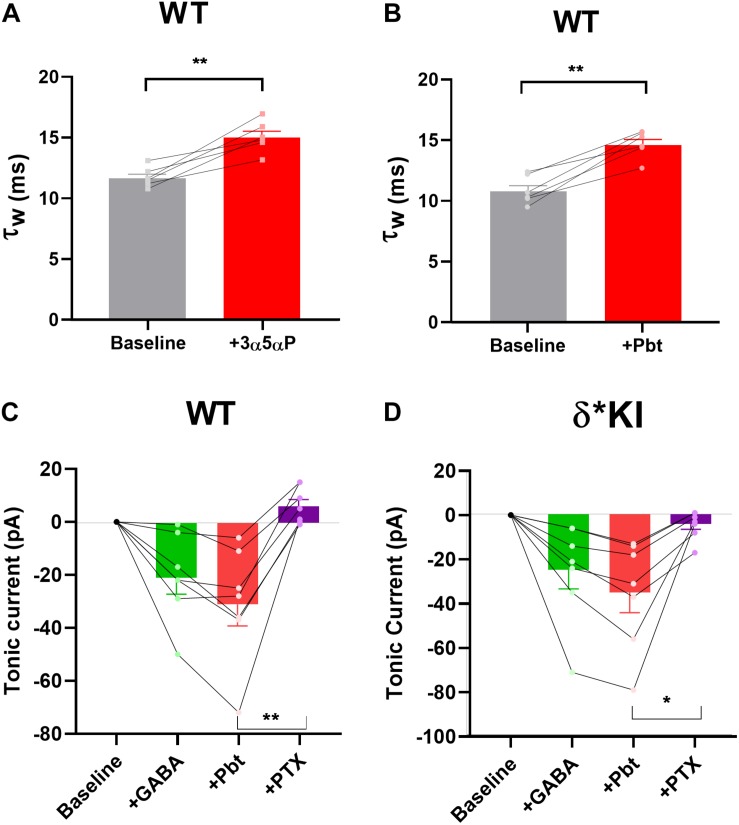
Pbt potentiates tonic current with exogenous GABA. **(A)** Replot of the effect of 100 nM 3α5αP on τ_w_ of sIPSCs (from Figure 1B) for comparison with Pbt effects. **(B)** 10 μM Pbt increased the τ_w_ of sIPSC decay in WT DGCs from 10.0 ± 0.5 ms to 14.7 ± 0.5 ms (*n* = 7). **(C)** Summary of GABA/Pbt generated current in indicated conditions in WT DGCs (*n* = 7). Tonic current under GABA, GABA+Pbt, and GABA+Pbt+50 μM PTX was −20.7 ± 6.2 pA, −30.7 ± 8.3 pA, and 6.3 ± 2.6 pA, respectively. **(D)** Summary of GABA/Pbt generated tonic current in indicated conditions in δ* KI DGCs (*n* = 7). Tonic current under GABA, GABA+Pbt, and GABA+Pbt+PTX was −25.3 ± 8.6 pA, −35.4 ± 9.3 pA, and −4.4 ± 2.4 pA, respectively. Pbt prolonged the decay of sIPSC (paired *t* test, *p* = 1 × 10^–3^). One-way ANOVA on tonic current showed a drug effect in WT (*F*(1.6,4.8) = 22.9, *p* = 4 × 10^–3^), and δ* KI (*F*(2.7,10.6) = 42.9, *p* < 1 × 10^–4^). PTX significantly decreased GABA/Pbt current in WT (Holm-Sidak, *p* = 2 × 10^–3^) and δ* KI (Holm-Sidak, *p* = 0.01).

We previously found that δ receptors do not contribute much to the overall current generated by a saturating GABA concentration and maximum δ receptor current was only 10–15% that of total current in DGCs (Sun et al., 2018). This difference in maximum contribution could mask δ receptor selectivity of neurosteroids. To address this possibility, we tested the PTX sensitivity of pentobarbital (Pbt), a broad-spectrum positive allosteric modulator, at a concentration chosen to mimic 100 nM 3α5αP on tonic current in the three genotypes. We found that 10 μM Pbt produced quantitatively similar sIPSC prolongation and increased tonic current to a similar degree as 100 nM 3α5αP (Figures 8A,B). In WT slices, 10 μM Pbt produced near 30 pA tonic current with exogenous GABA (Figure 8C), which is comparable to the GABA/3α5αP tonic current (Figures 4A,B). The GABA/Pbt tonic current was fully inhibited by PTX (Figure 8C), indicating that the entirety of the Pbt effect is mediated by GABA_A_ receptors. Importantly, in δ^∗^ KI slices, the GABA/Pbt generated tonic current was only partially blocked by PTX (Figure 8D), to a very similar degree as 3α5αP potentiated current (Figures 4C,D). Thus, we conclude that 3α5αP exhibits no more selectivity than the broad spectrum positive modulator, Pbt at an equivalent concentration.

## Discussion

In this study, we used subunits engineered to resist PTX antagonism to revisit the selectivity of neurosteroids on δ GABA_A_ receptors in mouse hippocampal DGCs. Our approach allowed us to measure pharmacological actions on different receptor populations in the same cell. First, we verified that 3α5αP potentiated phasic inhibition of GABA_A_ receptors mainly through γ2 receptors, although we recently showed a δ receptor component to IPSCs (Sun et al., 2018). Second, we showed that 3α5αP augmented tonic inhibition with or without exogenous GABA through both δ and γ2 GABA_A_ receptors. Third, our results showed that even with the subunit-selective agonist THIP, both 3α5αP and THDOC acted non-selectively at δ and γ2 receptor populations. Finally, the quantitatively similar effect of Pbt on PTX-resistant receptors mediating tonic current emphasizes the broad spectrum nature of neurosteroid potentiation. Overall, using a sensitive methodology, our results revise a prevalent view of neurosteroid selectivity on δ GABA_A_ receptors and reveal the broad spectrum nature of neurosteroid-augmented tonic inhibition. Our results indicate that the psychotropic actions of neurosteroids are unlikely to arise from selectivity at δ GABA_A_ receptors, although we do not exclude psychotherapeutic effects via extrasynaptic receptors.

In this study, we used mice with PTX resistance in either δ or γ2 GABA_A_ receptors. Mice were generated with a knock-in/chemogenetic approach described in our earlier work (Sun et al., 2018). The advantage of our approach is that we are able to investigate the selectivity of neurosteroids within the same cell, and there is less opportunity than with genetic deletions for compensation or other secondary effects to influence results. A caveat is that the γ2 mutation leads to faster IPSCs (Sun et al., 2018) (Figures 1E,F). Nevertheless, the complementary results obtained from the two mutant lines help mitigate the possibility that changes secondary to the T6’Y mutation account for results. Both mutant mouse lines showed similar response as WT to GABA and neurosteroid application throughout our experiments (Figures 3–8). Thus it is unlikely that the non-selectivity of neurosteroids results from different pharmacological profiles caused by the mutations. We cannot entirely exclude the possibility that PTX sensitivity is different in the presence of neurosteroid compared with agonist alone, and that this sensitivity changes with the induced mutations. However, previous results have shown that positive allosteric modulators gate currents entirely sensitive to PTX (Thompson et al., 1996).

Because the focus of our study was pharmacological actions of neurosteroids, we did not revisit the receptor source of endogenous tonic current in the absence of neurosteroid. In fact, in our hands the DGC standing current is very small (present study) or undetectable (Sun et al., 2018). In principle, the modest contribution of δ receptors to 3α5αP potentiation of tonic current could reflect recruitment of γ2 receptors that are silent in the presence of ambient GABA alone. Alternatively, the lack of selectivity could represent amplification of both receptor types already activated by ambient GABA. Past evidence favors the idea that γ2 receptors, perhaps those coupled with α5, may mediate some tonic current in DGCs and other cell types (Glykys et al., 2008; Kasugai et al., 2010; Patel et al., 2016).

Neurosteroids potentiate the actions of ambient GABA at low modulator concentration but directly gate the channel at somewhat higher concentration (Callachan et al., 1987; Cottrell et al., 1987; Puia et al., 1990; Shu et al., 2004). Because some cells showed no evidence of measurable ambient GABA current (Figure 3), we cannot exclude the possibility that direct gating in the absence of GABA explains current generated by γ2 receptors in some cells (Shu et al., 2004). As noted above, however, it may be more likely that 3α5αP increased the effectiveness of very low concentrations of GABA present, which are ineffective at baseline. Despite the presence of a non-saturating δ-preferring THIP concentration in Figure 5, the effect on γ2 appears to dominate. This seems surprising, as 3α5αP will increase agonist potency at both receptor classes. The balance of effects may be explained by the comparatively large number of γ2 receptors. Nevertheless, the discrepancy between the lack of neurosteroid selectivity and the high degree of DS2 selectivity is striking (Figure 5).

Our motivation was mainly to understand recent therapeutic, pharmacological effects of neurosteroid administration (Gunduz-Bruce et al., 2019). 3α5αP is also an endogenous neurosteroid that may contribute to ongoing inhibitory tone. Estimates of endogenous concentrations vary, but non-pregnancy neurosteroid levels likely reach 30–70 nM in humans (Bixo et al., 1997). We cannot exclude the possibility that these concentrations of neurosteroid contributed to the “baseline” IPSC and tonic-current profiles in our studies.

Our data indicate that the steroid-generated current was mediated approximately equally by δ receptors and γ2 receptors. Our previous results suggest that at saturating GABA concentrations, δ receptor current in DGCs is only 10–15% of total current (Sun et al., 2018). Thus, 50% contribution to neurosteroid-generated tonic current could reflect preference for δ receptors. However, because Pbt quantitatively mimicked the effect of steroids, we propose that the outsized effect of neurosteroid on δ receptors is likely the result of the higher GABA affinity of δ receptors compared with γ2 receptors rather than true selectivity of δ receptors for neurosteroid or Pbt (Brown et al., 2002; Wohlfarth et al., 2002). The low ambient GABA concentration will preferentially recruit δ receptors, and active receptors are more strongly affected by the positive allosteric effects of the steroid (Brown et al., 2002; Wohlfarth et al., 2002).

Although we found that 100 nM 3α5αP was necessary to reliably potentiate both phasic and tonic inhibition (Figures 1–3), a previous study showed that as little as 10 nM THDOC generated selective δ receptor effects, with higher concentrations exhibiting less selectivity (Stell et al., 2003). However, another study showed δ selectivity of 3α5αP up to 1 μM (Carver and Reddy, 2016), and still another found increased selectivity of δ receptor effects up to 10 μM of the synthetic neurosteroid alphaxalone (Spigelman et al., 2003). Thus, selectivity has been observed over a wide range of concentrations. Behavioral results have suggested that anesthetic and anxiolytic effects of neurosteroids are reduced in δ-deficient animals (Mihalek et al., 1999). By contrast with these studies, our results, using a different approach, support the idea that at moderate concentrations, neurosteroids do not preferentially target δ receptors.

Neurosteroids and ethanol share a proposed direct link, which was the motivation for us to examine ethanol as a GABA_A_ receptor modulator. GABA_A_ δ receptors may act as a sensor of low concentrations of ethanol (Wallner et al., 2003; Wei et al., 2004; Olsen et al., 2007), and at least some of ethanol’s effects on GABA_A_ receptors are proposed to occur through altered neurosteroid synthesis (Sanna et al., 2004; Tokuda et al., 2011), offering an explanation for δ selectivity. Although some results have disputed the δ selectivity of ethanol (Borghese et al., 2006; Yamashita et al., 2006; Fleming et al., 2011), we hypothesized that δ^∗^ and γ2^∗^ mice would provide new tools to revisit this issue. Our results failed to reveal significant potentiation of GABA_A_ mediated tonic current in DGCs, so at least in this cell type under our conditions, the results do not allow us to comment on the selectivity of ethanol or on ethanol-induced steroidogenesis for δ receptors.

In summary, we have used genetic, pharmacological, and electrophysiological approaches to investigate the selectivity of neurosteroids on GABA_A_ receptors. The sensitivity of our approach supports a broad spectrum action of neurosteroids. Recently, 3α5αP and another neuroactive steroid, SGE-217, have been shown to have antidepressant effects (Kanes et al., 2017; Gunduz-Bruce et al., 2019) and 3α5αP (brexanolone) was approved for treatment of postpartum depression by the Food and Drug Administration. Our work here suggests that δ selectivity is unlikely to underlie the benefit. Other targets for neurosteroids likely remain to be discovered that differ from other GABA_A_ receptor modulators, such as barbiturates and benzodiazepines, to account for antidepressant benefit.

## Data Availability Statement

The datasets generated for this study are available on request to the corresponding author.

## Ethics Statement

The animal study was reviewed and approved by the Washington University IACUC (Institutional Animal Care and Use Committee) Committee.

## Author Contributions

XL, SM, and CZ contributed to the conception and design of the experiments, revised the manuscript critically for intellectual content, and approved it for submission. XL performed experiments and wrote the first draft of the manuscript.

## Conflict of Interest

CZ is a member of the Scientific Advisory Board of Sage Therapeutics. Sage Therapeutics had no role in the design of execution of these studies. The remaining authors declare that the research was conducted in the absence of any commercial or financial relationships that could be construed as a potential conflict of interest.
